# Connecting women who are diagnosed and treated for breast cancer to engage in physical activity: a two-arm randomized controlled trial

**DOI:** 10.1186/s13102-025-01131-4

**Published:** 2025-04-25

**Authors:** Ross M. Murray, Erin O’Loughlin, Jenna Smith-Turchyn, Angela J. Fong, Meghan H. McDonough, Daniel Santa Mina, Kelly P. Arbour-Nicitopoulos, Linda Trinh, Jennifer M. Jones, Jackie L. Bender, S. Nicole Culos‐Reed, Jennifer R. Tomasone, Madison F. Vani, Catherine M. Sabiston

**Affiliations:** 1 University of Toronto, 55 Harbord Street, Toronto, ON M5S 2W6 Canada; 2 Centre de Recherche du Centre Hospitalier de l’Université de Montréal (CRCHUM), Montréal, Canada; 3 McMaster University, Hamilton, ON Canada; 4 Rutgers University, New Brunswick, NJ USA; 5 University of Calgary, Calgary, AB Canada; 6 University Health Network, Toronto, ON Canada; 7 Queens University, Kingston, ON Canada

**Keywords:** Exercise, Connectedness, Dyad, Oncology, Social support

## Abstract

**Background:**

Women who are diagnosed and treated for breast cancer (WBC) encounter barriers engaging in adequate physical activity (PA). Pairing WBC with PA partners is a feasible approach to promote social support, potentially increasing PA levels. However, WBC may not perceive to have the expertise required to facilitate PA behavior. As such, providing access to a Qualified Exercise Professional (QEP) may help facilitate PA within dyads. To date, the impact of including a QEP in peer-based interventions remains unclear.

**Methods:**

A two-arm randomized controlled trial (*n* = 108) was designed to compare a virtual peer and QEP-supported intervention group (MatchQEP: *n* = 54) to a control group matched only with a peer (Match: *n* = 54) on moderate-to-vigorous PA (MVPA). The association between social support within peer dyads and MVPA, and the impact of partners’ level of MVPA on individual MVPA were also examined. Participants in the *MatchQEP* condition met with the QEP and their partner on Zoom once per week for 10 weeks. Those in the *Match* condition were encouraged to independently communicate with and support their assigned partner over 10 weeks. Analyses involved descriptive statistics, regression analyses, and Actor Partner Interdependence models.

**Results:**

Social support significantly related to MVPA, irrespective of intervention group. The addition of a QEP did not yield additional benefits in increasing MVPA levels. Actor-Partner Interdependence Models reveal that partners’ PA behaviors did not significantly impact individual MVPA levels.

**Conclusion:**

These findings underscore the significance of social support from partners in promoting MVPA among WBC, emphasizing the need for interventions focusing on supportive partner relationships. By leveraging social support between partners, interventions can better address the unique needs of WBC, ultimately improving their health and well-being.

**Trial registration:**

Connecting Breast Cancer Survivors for Exercise (C4E), Trial Registration Number: NCT04771975, 28/02/2020.

**Supplementary Information:**

The online version contains supplementary material available at 10.1186/s13102-025-01131-4.

Women who are diagnosed and treated for breast cancer (WBC) often experience negative health consequences such as cancer-related fatigue, depression, and anxiety, which may stem from the cancer itself or its treatment. These health consequences are also barriers to engagement in physical activity, and the relationship is circular in that low levels of physical activity can exacerbate health challenges and is independently associated with an increased risk of cancer recurrence [1–4]. Consequently, most WBC do not meet the WHO recommended aerobic physical activity guidelines of 150 min of moderate intensity physical activity per week or 75 min of vigorous intensity physical activity per week [5–9]. Furthermore, evidence-based messages suggest individualized physical activity programs designed with qualified exercise professionals and in supportive environments are important for health outcomes [8]. Yet, WBC often lack access to resources that facilitate social support for physical activity [10–13]. In support of the evidence, key ways to offer social support for physical activity include matching WBC with similar others, mentors, and qualified exercise professionals (QEP).

Social support positively relates to physical activity behavior among WBC [14–16]. Within the context of physical activity, researchers typically examine social support as the provision of tangible (e.g., financial assistance, products, time), informational (e.g., advice, feedback), emotional (e.g., comfort, care), and esteem (e.g., confidence, reassurance) support [17]. These support provisions can come from a variety of sources (e.g., family, friends, colleagues, health care workers), however, in many instances support provisions may be unavailable for WBC, especially those in hard-to-reach populations, such as WBC living in rural areas [12]. Further, strategies to facilitate social support for physical activity can be resource intensive, requiring financial support and expert training. As such, social support offers significant potential to increase physical activity levels.

An acceptable and feasible approach to facilitate social support in physical activity interventions is to match individual WBC in dyads [18]. These types of interventions aid in social facilitation (i.e., viewing others being physically active beyond a cancer diagnosis, receiving encouragement from others who are similar, receiving reminders regarding physical activity through communication, education and information-sharing with peers) which may increase self-efficacy, motivation and confidence in being physically active [19]. Further, offering virtual peer support dyadic interventions may help diminish barriers including fear of viral infection, distance, time, and cost [20–22]. Although virtual home-based programs may lower adherence to the intervention [23], they are often more accessible and less resource intensive than programs offered in different types of settings. Further, they may increase the likelihood of sustaining physical activity levels attained once the intervention is ended, especially if peer-support is maintained [24]. Previous peer intervention research among WBC has adopted a mentoring approach, whereby individuals with experience navigating physical activity after a cancer diagnosis mentor more recently diagnosed individuals [25–28]. These interventions match trained mentors with a peer, whereby there is differing authority regarding physical activity [25, 26, 28]. The interventions are delivered by individuals who have experience, knowledge, resources, and training to provide counsel and empathy in facilitating physical activity within their peers [29]. However, the process of training, matching, and monitoring peer mentoring dyads demands substantial resources and establishes a power dynamic wherein the mentor holds greater authority within the pairing and may be deemed too professional [29]. In contrast, online peer-matching physical activity systems have been developed specifically for WBC to facilitate social support during physical activity (see ActiveMatch [activematch.ca]). Herein “peer-matching physical activity systems” refers to matching peers who have equivalent roles and authority within a dyad. Generally, dyadic interventions may be as successful because peers share experiences and a common goal in striving to increase their physical activity [30]. As such, within these dyads, the physical activity of Peer 1 may facilitate physical activity in Peer 2 [31, 32] as the provision of social support and as contagion effects. Although peer-to-peer support is promising, matching WBC with peers does not address lack of access to qualified physical activity guidance and appropriate programming. Having a behavioral support program may increase adherence to physical activity guidelines from 46 to 90% [33–35], at least while support is present from a qualified exercise professional (QEP). However, it remains unclear whether support from a QEP promotes sustained physical activity post-intervention. For example, Westphal et al., [23] randomized breast cancer patients to either a counseling group, or a counselling group with supervised physical training. The supervised group attained higher fitness levels, but slight declines in fitness were observed in both groups 24-weeks post-intervention. The authors suggested that key issues in this domain are how to increase adherence to newly attained physical activity levels in the longer-term and the feasibility of such interventions in terms of cost. Within the current study, two strategies were used to respond to these suggestions. First, to address the barriers faced by women with breast cancer (WBC) in engaging in physical activity, the inclusion of a Qualified Exercise Professional (QEP) was hypothesized to enhance outcomes by combining behavioral support with accountability. This behavioral approach aimed to foster sustainable engagement in physical activity rather than solely delivering supervised programming and is consistent with recommendations for physical activity programming for people with cancer [8]. The second strategy implemented in the current study was to partner WBC to provide sustainable social support. As such, the purpose of the current study was to examine the effects of a peer and QEP-support virtual intervention group (labelled MatchQEP), compared to a control group of WBC who were matched with a peer, but not a QEP (labeled Match) on moderate-to-vigorous physical activity (MVPA). Additionally, the association between social support within dyads and MVPA was examined. Finally, using actor-partner interdependence modelling, the impact of partners’ level of MVPA on the other peer’s MVPA was tested. All aims were explored for both self-report and device-measured MVPA. We hypothesized that WBC in the MatchQEP would increase their physical activity compared to WBC in the Match group. Beyond the effect of the intervention, we also hypothesized that WBC who perceived higher levels of support from their partner would engage in more physical activity compared to WBC who perceived less support. Finally, we hypothesized there would be a significant contagion effect, whereby the PA women’s partners engaged in would significantly relate to the PA they engage in. These hypotheses were examined across both self-report and device-measured physical activity where the effects were expected to be similar.

## Method

### Study design and participants

Approval for this two-arm randomized controlled trial was provided by The University of Toronto’s Human Research Ethics Unit (protocol #00038665). This study adheres to the CONSORT guidelines [36]. Recruitment was completed through digital materials such as e-mails to community centers and social media posts which signaled interested participants to contact the study team. Participants were entered and matched into the study by rolling recruitment which took approximately 8 weeks. Data were collected between August 2021 and June 2022. Participants were 108 women randomly allocated to either the intervention (MatchQEP, *n* = 54) or control (Match, *n* = 54) group prior to baseline assessment, after being matched with their peer using specific match criteria [37]. Sample size calculation was carried out based on actor-partner interdependence modelling (APIM) power analysis for indistinguishable dyads. Specifically, the alpha was set to 0.05 and the sample size estimate is the smallest number of dyads required to detect the effect when power is at least 0.8. Recruitment was ongoing until the sample size calculated for power was complete (*n* = 108). To be included in the study, participants were (1) English-speaking female WBC; (2) diagnosed with primary stage 0–IV breast cancer, at any stage of treatment, or completed treatment; (3) living in Canada; (4) aged 18 years or older; (5) medically cleared for physical activity; (6) connected to the internet using any device (e.g., computer, tablet or smartphone; preferably with webcam); (7) engage in less than 150 min of moderate intensity physical activity or 75 min of vigorous physical activity per week (assessed using the Godin Leisure Time Exercise Questionnaire [38]). Individuals who had any contraindications to physical activity or planned surgery of any kind were excluded from the study.

Dyads were randomized (1:1) to the intervention (MatchQEP) or control (Match) group prior to baseline assessment (after the demographic questionnaire was completed). Randomization was conducted by a Ph.D. student external to the research team using Randomizer.org. Matching was done based on previous work [39], using the following criteria in order of importance: (1) Age, (2) Time zone, (3) Family situation (e.g., married/divorced, number and age of children living at home), (4) Current exercise volume. The randomization to study arms was concealed, but the matches were not (as the matching process used predefined criteria). All participants were provided with evidence informed tips and strategies for supporting a physical activity partner [40], and a one-page infographic highlighting current physical activity guidelines for cancer survivors [8]. All participants were also given a Fitbit wearable activity tracking device to be worn during the assessment periods and optionally during the non-assessment weeks. Based on initial research findings exploring physical activity peers among WBC [16], partners were introduced to one another by a research assistant via Zoom in an initial conference call prior to beginning the study. Data were collected at four time points: Baseline (prior to introduction to partners and QEP), post 10-week intervention, follow up post tapering (i.e., 14-weeks after baseline), and longer-term follow-up (i.e., 24-weeks after baseline).

#### Intervention condition

In addition to the physical activity information provided to all participants, those in the *MatchQEP* (experimental) condition received tailored program sessions delivered by a QEP. The lead QEP was a Ph.D. in Exercise and Health Psychology with certifications in personal training and group fitness and advanced specialized training in exercise programming for individuals living with and beyond cancer. Participants in the *MatchQEP* condition met with the QEP and their partner on Zoom once per week over the course of 10 weeks. Each 60-minute session led by a QEP, designed to address both the behavioral and logistical barriers to physical activity. These sessions included personalized discussions on topics such as goal-setting, building and maintaining social support, overcoming barriers, and habit formation, supplemented by a review of past activity and accountability for planned goals. This intervention was theoretically grounded in the Behavior Change Wheel framework [40], ensuring evidence-based approaches to sustainable physical activity. The COM-B model, a core part of the Behavior Change Wheel, guided the design of the intervention to address participants’ capability (e.g., skill-building and progression), opportunity (e.g., fostering social support and creating accessible goals), and motivation (e.g., self-reflection and positive reinforcement) for behavior change. Each session included a past-week accountability check-in and goal-setting for the upcoming week, tailored to participants’ cancer-related circumstances and physical activity levels. See Smith-Turchyn et al. [37] for more details. The goal of the QEP delivered physical activity program was to increase participants MVPA to a minimum of 150 min per week [8]. Aligned with recommendations, the sessions were tailored to individuals’ personal circumstances such as cancer related characteristics, side effects, fitness level, and available resources [8]. The intervention was 10 weeks in length, and participants were also offered a four-week tapering period following the intervention period. During tapering period, the QEP was available to participants for specific personal questions.

#### Control condition

Participants in the *Match* (control) condition were provided with a Fitbit and encouraged to independently communicate with and support their assigned partner over the intervention period. Participants in this group did not have contact with the QEP, but 14 weeks after the intervention period (encompassing the 4-week tapering period and 10-week follow-up timeline), they were offered a single session physical activity consultation.

### Measures

#### Self-report MVPA

Volume of MVPA was the primary outcome and evaluated through the modified Godin Leisure Time Exercise Questionnaire, a reliable and valid self-report tool [38, 41]. Participants self-report the frequency and duration of vigorous and moderate aerobic activities during the last 7 days (one week). A single additional item was added to explore resistance training frequency and duration in the last week. Responses regarding moderate and vigorous activities were aggregated as total volume of MVPA. This measure was also used post-tapering (i.e., 14 weeks) and follow-up (i.e., 24-weeks) post-baseline.

#### Device-measured MVPA

Volume of device-measured MVPA was a secondary outcome and evaluated using the Fitbit Inspire 2 accelerometer. Previous research indicates strong adherence to Fitbit usage among individuals diagnosed with cancer [42, 43], and Fitbit physical activity data shows a strong correlation to Actigraph measures within this population [42]. Fitbit determines active minutes using metabolic equivalents (METs), classifying activity into light physical activity (LPA; 1–3 METs) and moderate-to-vigorous physical activity (MVPA), with moderate activity ranging from 3 to 6 METs and vigorous activity exceeding 6 METs. Fitbit devices were distributed to WBC at the beginning of the study, with a requirement to wear them continuously for seven consecutive days during the four primary data collection periods. Baseline MVPA was assessed as the total amount of moderate and vigorous physical activity engaged in over the week before beginning the study. Post-intervention MVPA was assessed as the total amount of MVPA engaged in during the last week of the study. The follow up assessments were measured as the total amount of MVPA engaged in during the tapering period (14-weeks post-baseline) and follow-up (24-weeks post-baseline). The total duration of device wear and corresponding data were retrieved from the online Fitbit database accessed through each participant’s unique and deidentified study Fitbit account. Participants kept the Fitbit device post-intervention.

#### Social support

Physical activity-related social support was assessed as a secondary outcome after the intervention period, utilizing a subset of 4 items derived from the Social Support Survey in Sport [44]. The stem of the questionnaire was modified to align with the partner-based model of social support employed in this study, inviting participants to indicate the extent to which their partner provided tangible (resources aiding physical activity), emotional (empathy towards physical activity-related challenges), informational (guidance on physical activity benefits WBC), and esteem (encouragement to physical activity) support. Responses were recorded on a scale ranging from 1 (not at all) to 7 (a lot). Items were aggregated and the mean of the 4 items was used as a main social support variable. Previous research has established the validity and reliability of the Social Support Survey in Sport [44] and has been used to examine support in WBC [16].

#### Demographics

Age in years, ethnicity, marital status, children, education, employment status, weight, stage of cancer, treatment status, treatment type, and years since diagnoses were reported in an online demographic questionnaire. Individuals’ status whether they met physical activity guidelines at each measurement occasion were calculated based on their self-report and device-measured physical activity scores.

### Analysis

#### Descriptive analysis

Data used to describe participants were collected and reported using means, standard deviations, and/or frequencies. Data were calculated and reported across all four time points (baseline, 10-week post-intervention, 14-week follow up, and 24-week follow up). The extent to which individuals met physical activity guidelines were described.

#### Intervention group and social support as predictors of MVPA

Regression analyses were run in R using the lmer function within the lme4 package [45]. Multilevel linear models were used to examine the effects of the intervention (MatchQEP vs. Match) on self-reported and device-measured MVPA at the end of the 10-week intervention. Participants completed the study in partners, meaning data on MVPA were not independent. As such, partner was included in the model as a random effect. There was no expectation that associations between group and MVPA would vary dependent on dyad, as such, a random intercept, fixed slope model was conducted. Covariates (participant age, pre-intervention MVPA) and the predictor variables (intervention group: MatchQEP vs. Match, and perceptions of social support) were included as fixed effects. Age was included to control for variations in physical activity typically observed across different age groups and baseline MVPA levels to assess changes in MVPA following the commencement of the study. Separate models were run examining post-intervention self-reported MVPA and post-intervention device measured MVPA as outcome variables. Predictors of self-report and device measured MVPA were also examined at the 14-week and 24-week follow ups.

#### Contagion effects

Actor-partner interdependence models (APIMs) were used to determine actor and partner time lagged effects of MVPA in study participants across all four time points. Missing data were handled using full information likelihood (FIML). Separate models were used to test these associations using self-report and device measured MVPA. Partners in this study were matched based on pre-existing matching criteria whereby neither individual was assigned a specific role within the dyad (i.e., no mentor/mentee roles), and as such, dyads were treated as indistinguishable [46]. All four measurement occasions (baseline, post-intervention, 14-week and 24-week follow up) were used to examine the effect of physical activity across partners over time.

## Results

### Descriptive results

**Fig. 1:**
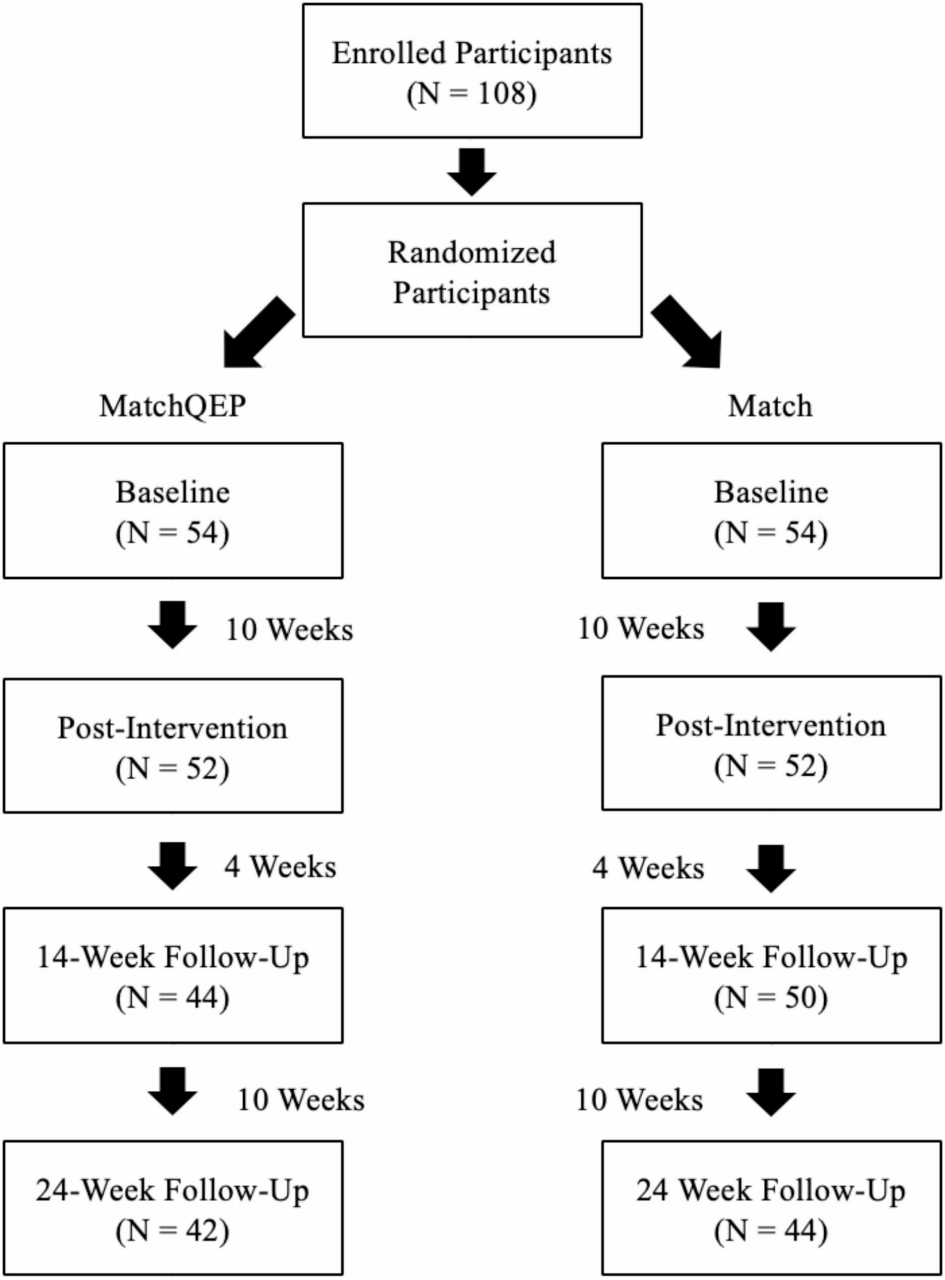
Participant flow diagram for the randomized controlled trial. A total of 108 participants were enrolled and randomized into two groups: *MatchQEP* (*n* = 54) and *Match* (*n* = 54). Each group underwent baseline assessment, followed by a 10-week intervention. Post-intervention assessments were conducted, with follow-ups at 14 weeks and 24 weeks. Participant retention numbers are provided at each stage

**Fig. 2:**
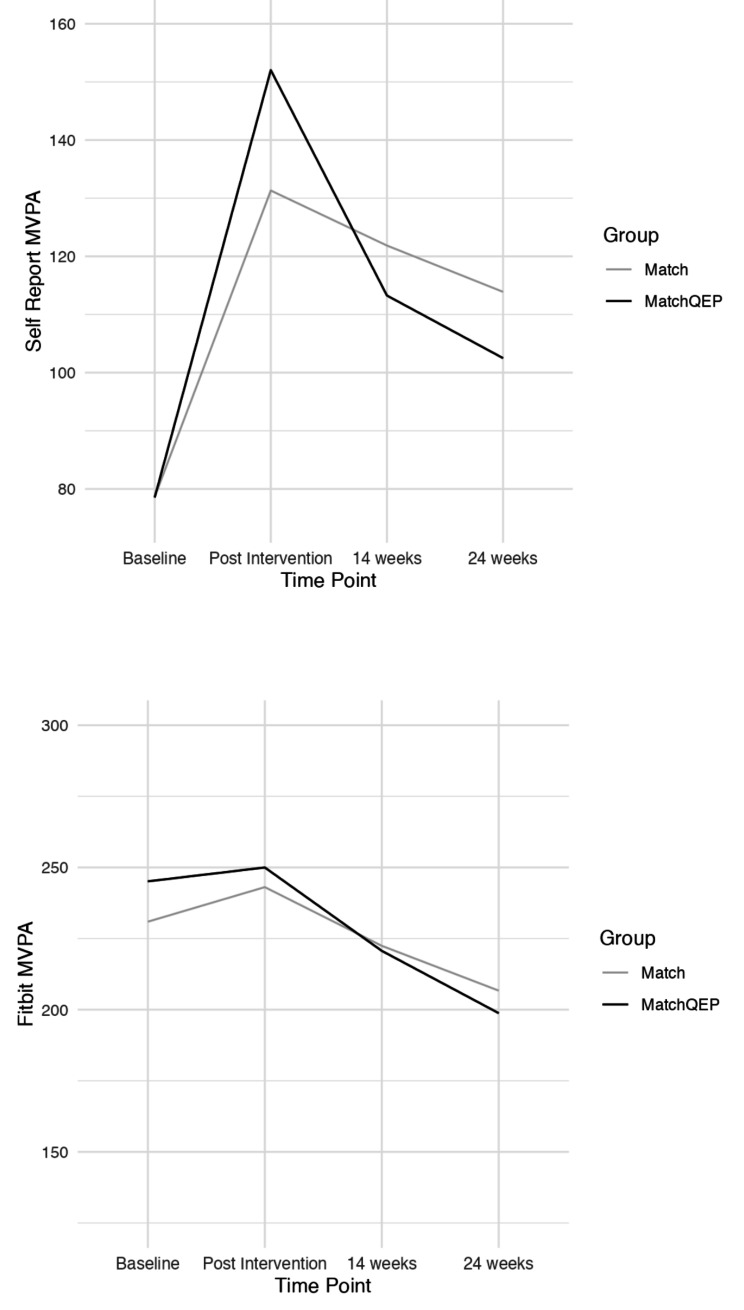
MVPA at each measurement occasion for the *Match* and *MatchQEP* Note: MVPA = moderate to vigorous physical activity, QEP = qualified exercise professional

In the *MatchQEP* group, participants attended on average 8.4 of the 10 possible sessions (range = 3–10). Survey completion dropped throughout the study, with 4 participants (50% MatchQEP) not completing the post intervention survey, 9 with incomplete 14-week data (67% MatchQEP), and 9 with incomplete 24-week follow up data (67% MatchQEP). See Consort Diagram (Fig. 1). Examination of linearity, normality, and homoscedasticity revealed no violations of assumptions. Table 1 displays participant characteristics and Table 2; Fig. 2 present self-report and device-measured MVPA across both study conditions. Both the *Match* (*t*(48) = 2.80, *p* =.007) and *MatchQEP* (*t*(49) = 4.19, *p* <.001) groups showed significant increases in MVPA from baseline to post-intervention. The change from baseline to post-intervention was not significantly different between the groups (*t*(97) = -1.00, *p* =.320). Changes in post-intervention MVPA and 14- and 24-week MVPA were not significant within or between the groups (*p* =.30 to 0.88). These findings indicate that the changes in MVPA were similar between groups over time. Based on the Fitbit data, both groups remained relatively stable in their MVPA levels during the intervention period, however declined at 14-week follow up, and again at the 24-week follow up. These declines were not statistically significant (all *p’*s > 0.05).

For social support, the *MatchQEP* group showed significant declines from post-intervention to the 14-week follow-up (*t*(40) = 3.65, *p* <.001) and from the 14-week to 24-week follow-up (*t*(43) = 3.75, *p* <.001). In contrast, the *Match* group showed smaller, non-significant declines in social support during these periods: from post-intervention to 14-week follow-up (*t*(49) = 1.79, *p* =.080) and from the 14-week follow-up to the 24-week follow-up (*t*(47) = 2.00, *p* =.051). For social support subscales, tangible support declined significantly from post-intervention to the 4-week follow-up for *Match* (*t*(49) = 2.11, *p* =.040) and *MatchQEP* (*t*(47) = 2.10, *p* =.041) and continued to decline significantly from 14 weeks post baseline to 24 weeks post baseline (*t*(47) = 2.97, *p* =.005; *t*(45) = 3.12, *p* =.003). Esteem support followed a similar pattern, with significant declines from post-intervention to 14 weeks (*t*(49) = 2.23, *p* =.030) and from 14 weeks to 24 weeks (*t*(47) = 2.11, *p* =.040) in both groups. For emotional support, *MatchQEP* experienced a significant drop from post-intervention to 24 weeks post baseline (*t*(46) = 2.90, *p* =.005), while the decline in *Match* was smaller and non-significant (*t*(49) = 1.87, *p* =.068). Informational support also declined significantly from post-intervention to 24 weeks post baseline for both *Match* (*t*(49) = 2.26, *p* =.027) and *MatchQEP* (*t*(47) = 2.26, *p* =.027). These findings suggest that while both groups experienced declining social support over time, the reductions were more pronounced and statistically significant in the *MatchQEP* group, particularly for emotional support.

**Table 1:** Sociodemographic and clinical characteristics of WBC according to intervention group

	Match group (*n* = 54)	MatchQEP group (*n* = 54)
Age (M(SD))	53.4 (13.0)	48.4 (9.4)*
Ethnicity (% White)	80	74
Married or living with partner (%)	70	74
Do you have a child or children (% yes)	83	69
Highest education completed (% ≥ university)	69	65
Currently employed, full- or part-time (% yes)	65	54
Weight (M(SD); range in kg)	74.5 (15.9)	75.7 (17.4)
Breast cancer stage diagnosis (% Stage ≤ II)	70	67
Completed treatment (% yes)	72	74
Breast cancer treatment (%)		
Lumpectomy	39	67*
Single or double mastectomy	57	44
Chemotherapy	76	67
Radiotherapy	70	72
Hormonal therapy	52	41
Years since diagnosis (M(SD))	5.6 (4.4)	4.6 (4.7)

Note: * significant difference between the groups based on t-tests and univariate chi-square tests

**Table 2:** Means and standard deviations for self-report and Fitbit-measured MVPA levels and social support. MVPA is recorded in average minutes per week

		Baseline M(SD)	Post-intervention M(SD)	14-week follow up M(SD)	24-week follow up M(SD)
Self-report	Match	79 (82)	131 (106)^†^	122 (106)	114 (114)
	MatchQEP	79 (85)	152 (117)^†^	113 (104)	102 (107)
Fitbit	Match	231 (184)	243 (198)	222 (217)	207 (188)
	MatchQEP	245 (158)	250 (194)	121 (179)	199 (179)
Tangible	Match	-	2.10 (1.69)	1.73 (1.47)^†^	1.39 (1.20)^†^
	MatchQEP	-	2.10 (1.77)	1.77 (1.80)^†^	1.21 (0.83)^†^
Emotional	Match	-	3.51 (2.34)	3.27 (1.98)	3.02 (2.23)^*†^
	MatchQEP	-	4.22 (2.26)	2.91 (2.38)	1.85 (1.69)^*†^
Informational	Match	-	2.41 (1.81)	2.18 (1.53)	2.12 (1.92)^*†^
	MatchQEP	-	2.88 (2.15)	1.95 (1.80)	1.38 (1.23)^*†^
Esteem	Match	-	3.63 (2.16)	3.04 (1.47)^†^	2.82 (2.20)^*†^
	MatchQEP	-	4.04 (2.20)	2.75 (2.29)^†^	1.87 (1.80)^*†^
Total social support	Match	-	2.91 (1.70)	2.55 (1.51)^*^	2.33 (1.65)
	MatchQEP	-	3.31(1.73)	2.34 (1.87)^*^	1.57 (1.71)^*†^

Note: Self-report MVPA ranged between 0 to 650 min per week. Fitbit MVPA ranged between 7 to 947 min per week. Social support scores ranged between 1–7

^†^indicates a significant difference (*p* <.05) from the previous time point

* indicates a significant difference (*p* <.05) between Match and MatchQEP group at that time point

### Physical activity guidelines

**Fig. 3:**
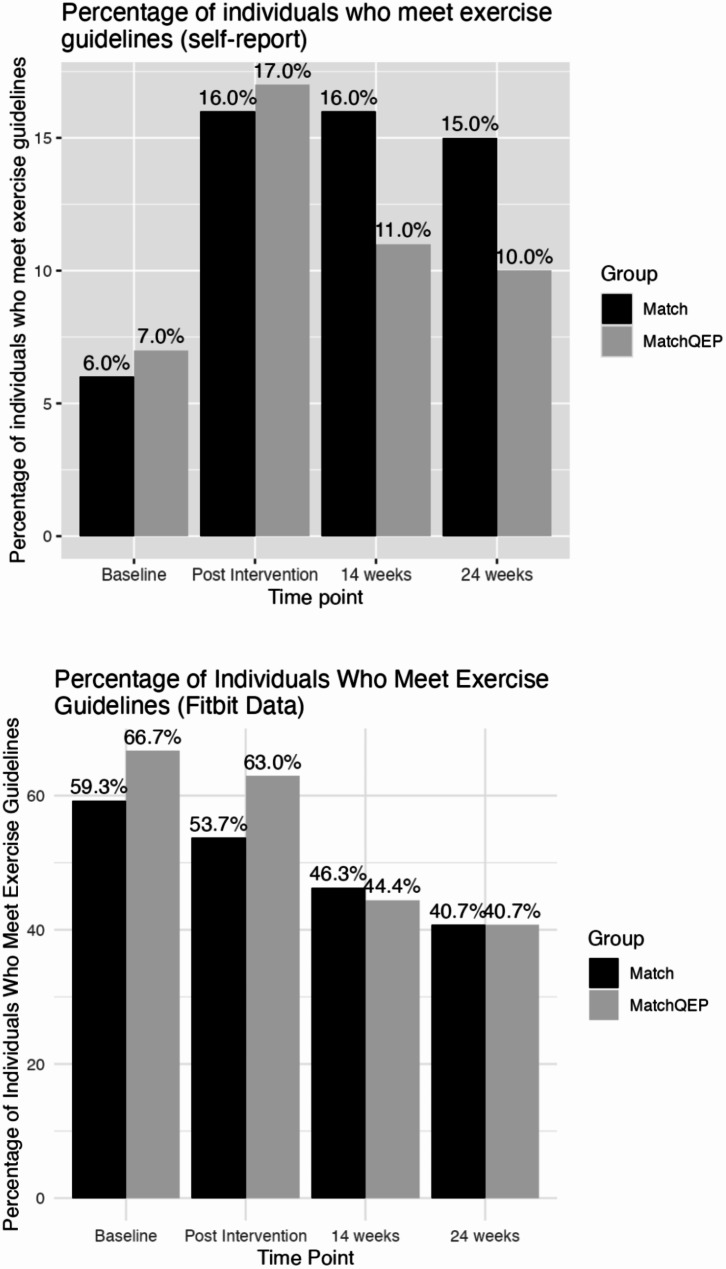
Percentage of individuals who met exercise guidelines (over 150 min of MVPA per week) in *Match* and *MatchQEP* groups. The top image is self-report data, the bottom image is Fitbit data

Although eligibility criteria specified individuals should engage in less that 150 min of MVPA per week, 13 individuals reported engaging in more than 150 min per week when completing baseline measures. These participants were retained in the analyses. The percentage of WBC meeting MVPA guidelines across the study period are graphed in Fig. 3. After the intervention, more participants self-reported meeting physical activity guidelines in both groups. At the 14-week follow up, 11% of individuals met physical activity guidelines in *MatchQEP* group, compared to 16% in the *Match* group. At the 24-week follow up 11% met guidelines in the *MatchQEP* group compared to 15% in the *Match* group. Chi square tests indicated these group differences were not statistically significant at any of the time points (all *p*’s > 0.05). In the Fitbit data, the number of individuals meeting MVPA guidelines decreased in both groups across the study, but there were no significant group differences (all *p*’s > 0.05).

### Regression models

#### Intervention and social support as predictors of self-report MVPA

The analytic sample was comprised of individuals with complete data in all study variables (*N* = 86, K = 49). The intra-class correlation coefficient for self-reported MVPA was 0.00, indicating there was no systematic variance in physical activity levels between the dyads. Analysis of covariates indicated that baseline self-report MVPA levels (*b* = 0.19, *SE* = 0.14, *p* =.17) and participate age (*b* = 0.71, *SE* = 1.09, *p* =.52) did not significantly correlate with self-reported MVPA post-intervention. When controlling for the effects of these covariates and intervention group assignment, social support was significantly associated with MVPA, whereby higher levels of support were associated with higher levels of MVPA (*b* = 3.6, *SE* = 1.9, *p* =.05). Finally, the intervention condition (Match vs. MatchQEP) did not significantly relate to self-report MVPA (*b* = 16.5, *SE* = 24.9, *p* =.51). Self-report baseline MVPA was a significant predictor of self-report MVPA at the 14-week (*b* = 0.36, *p* =.012) and 24-week (*b* = 0.35, *p* =.014) follow up data collections. Intervention group, participant age, and partner support were not significant predictors of MVPA at 14- or 24-week follow ups (*p*s > 0.05).

#### Intervention and social support as predictors of device-measured MVPA

There was no systematic variance between dyads (ICC = 0.00). Device measured MVPA before the intervention was significantly associated with post-intervention MVPA (*b* = 0.66, *SE* = 0.10, *p* <.01), but participant age was not (*b* = -2.15, *SE* = 1.5, *p* =.18). After accounting for covariates, there was no significant association between social support and device-measured MVPA (*b* = 2.9, *SE* = 2.7, *p* =.29) or intervention grouping (Match vs. MatchQEP) in device measured MVPA (*b* = -6.19, *SE* = 37.86, *p* =.87). Device measured baseline MVPA was a significant predictor of MVPA at the 14-week (*b* = 0.58, *p* <.01) and 24-week (*b* = 0.56, *p* <.01) follow ups. Intervention group, participant age, and partner support were not significant predictors of MVPA at 14- or 24-week follow ups (*p*s > 0.05).

### Actor partner interdependence models

#### Self-report MVPA

**Fig. 4:**
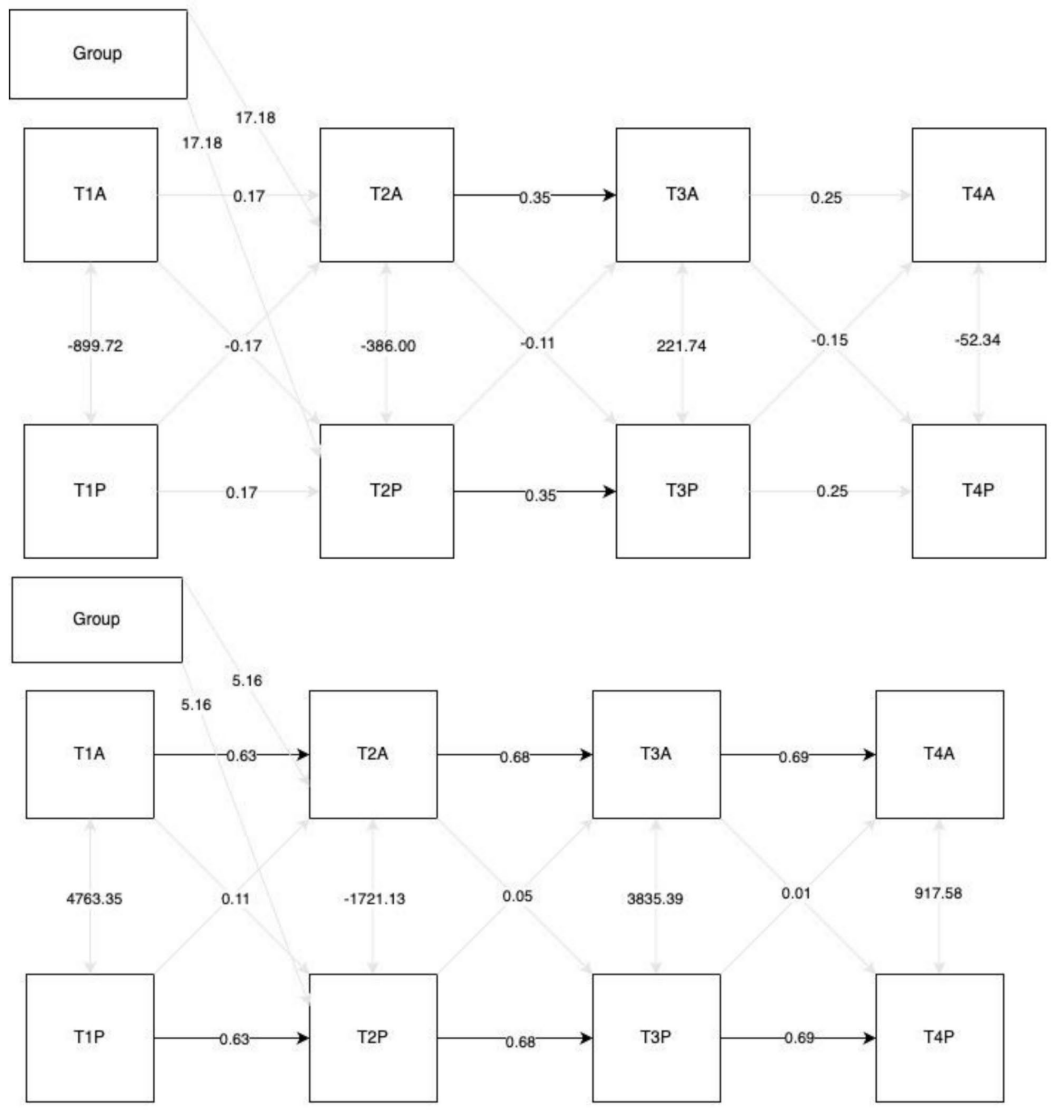
Actor partner interdependence model. Top figure represents self-report data, bottom figure represents Fitbit data Note: T1 = baseline, T2 = post intervention, T3 = 14-week follow-up, T4 = 24-week follow up. *P* = Partner, A = Actor. Black lines represent significant pathways

When included in the actor-partner interdependence model (Fig. 4), there was no effect of intervention group on self-report MVPA after the intervention (*b* = 17.18, *SE* = 22.40, *p* =.77). There was also no significant effect of actors’ self-report MVPA pre-intervention on actors’ self-report MVPA at post-intervention (*b* = 0.16, *SE* = 0.12, *p* =.18). The was no significant association between actors’ self-report MVPA pre-intervention on partners’ self-report MVPA post-intervention (*b* = − 0.18, *SE* = 0.13, *p* =.17). There was a significant effect of actors’ self-report MVPA post-intervention on actors’ self-report MVPA at the 14-week follow up (*b* = 0.39, *SE* = 0.15, *p* =.01). This association indicates that the physical activity level of individuals after the intervention had a significant impact on their physical activity level 4-weeks later. The was no significant association between actors’ self-report MVPA post-intervention on partners’ self-report MVPA at the 14-week follow up (*b* = 0.02, *SE* = 0.20, *p* =.91), and no significant partner associations from the 14-week follow up to the 24-week follow up (*b* = 0.25, *SE* = 0.18, *p* =.16). This indicates the amount of MVPA individuals self-reported did not influence on the amount of MVPA their partner self-reported.

#### Device-measured MVPA

There was no effect of intervention group on self-report MVPA post-intervention (*b* = 5.16, *SE* = 33.2, *p* =.87). There was a significant effect of actors’ device-measured MVPA pre-intervention on actors’ device-measured MVPA post-intervention (*b* = 0.63, *SE* = 0.18, *p* <.01), indicating that the amount of MVPA participants were doing before the intervention significantly impacted the amount of MVPA they were doing after the intervention. There was no significant association between actors’ device-measured MVPA pre-intervention on partners’ device-measured MVPA post-intervention (*b* = 0.11, *SE* = 0.09, *p* =.25), meaning partners’ device-measured MVPA levels did not influence one another. There was also a significant effect of actors’ device-measured MVPA after the intervention on actors’ device-measured MVPA at the 14-week follow up (*b* = 0.68, *SE* = 0.11, *p* <.01), indicating that the amount of MVPA participants were doing after the intervention significantly impacted the amount of MVPA they were doing 4-weeks later. There was no significant association between actors’ device-measured MVPA after the intervention on partners’ device-measured MVPA at the 14-week follow up (*b* = 0.05, *SE* = 0.06, *p* =.40). There was a significant actor association between the 14-week and 24-week follow up (*b* = 0.69, *SE* = 0.08, *p* <.01), but no significant partner associations (*b* = 0.01, *SE* = 0.05, *p* =.12). These results indicate that device-measured MVPA was impacted by the amount of MVPA individuals were doing at the previous time point, but not by the amount of MVPA their partner was doing at the previous time point.

## Discussion

This study examined the effect of partner-based peer support during a physical activity intervention for WBC who either received QEP sessions for 10 weeks (MatchQEP) or did not (Match control group). The results indicated that self-reported physical activity increased across both the *Match* and *MatchQEP* conditions from baseline to post-intervention; however, there was no significant difference between groups. Regardless of the assigned condition, social support received from women’s physical activity partners significantly predicted higher levels of self-reported MVPA. While participants’ baseline level of MVPA was associated with MVPA post intervention and 4-weeks later, the amount of physical activity partners engaged in did not significantly relate to the partner MVPA. These non-significant associations were consistent across both self-report and device-measured physical activity.

Participants in both conditions showed increased self-reported MVPA across the trial. However, both groups experienced declines post-intervention, with the MatchQEP group exhibiting more pronounced decreases, suggesting that the removal of resources (i.e., the QEP support) may lead to regression towards pre-intervention activity patterns. This finding is consistent with meta-analyses indicating that the inclusion of certain strategies, such as QEP, may increase short term physical activity, but inhibit lasting change [47]. As such, when examining the descriptive changes across this study, omitting the QEP and just providing peer support may be a more resource effective strategy to facilitate physical activity change.

Contrary to hypotheses, there was no significant difference in the amount of self-report MVPA, or device-measured MVPA, between the two conditions (those partnered solely with another WBC versus those partnered with both a WBC and a QEP) at any assessment. This finding suggests that, despite previous research indicating the efficacy of adding a QEP to provide a behavioral support program [48], the addition of a QEP did not yield additional benefits in terms of increasing sustainable MVPA. As such, even though guidelines for physical activity among individuals diagnosed with cancer underscore the criticality of supervised physical activity programming [8, 49, 50], the QEP may not offer additional benefits to the volume of MVPA over and above the social support provided by peer physical activity partners.

Regardless of whether women were partnered solely with another WBC or with both a WBC and a QEP, higher levels of social support from their partner were associated with higher levels of self-reported MVPA. This finding aligns with previous research indicating physical activity partners can positively impact partners’ physical activity level [16] and underscores the importance of interpersonal relationships and the supportive environment provided by partners in facilitating physical activity engagement among WBC. Consistent with research in the sport context, social support is more effective when provided by someone the support receiver identifies with [51]. In the current study, all participants have experienced breast cancer and were partnered with individuals with similar characteristics based on age, time zone, and history of physical activity [39]. This partnering may create an underlying social identity within the dyad, which may facilitate the association between social support and physical activity behavior. However, the inclusion of a QEP within these partnerships may have hindered the sense of social support or identity between physical activity partners. Future research is needed to examine whether adding a leader or professional within small groups negatively impacts a sense of autonomy and group dynamics, possibly due to participants becoming overly dependent on the leader, thereby weakening peer-to-peer interactions and shared identity.

Social support can influence how individuals perceive their physical activity levels, which may explain discrepancies between self-reported and device-measured MVPA. By enhancing self-efficacy and self-regulation, social support can encourage individuals to view routine movements, such as gardening, as part of their exercise routine [52]. This cognitive reframing may lead to inflated self-reports of MVPA, even when objective measurements do not show corresponding increases. Since perceptions of activity, rather than actual movement, can significantly impact health outcomes [53, 54], future research should explore how social support shapes subjective activity assessments and whether this moderates the relationship between self-reported and device-measured MVPA.

Social support declined over time, consistent with research showing that intervention-related support often diminishes without continued reinforcement [55]. *MatchQEP* experienced a steeper decline in emotional support compared to *Match*, suggesting that having structured external support may not be as effective in sustaining long-term perceptions of support as peer-based partnerships. Tangible and informational support also decreased, aligning with evidence that instrumental support wanes as obligations lessen [56], while esteem support declined without ongoing reinforcement [57]. The smaller decline in the *Match* group suggests that having participants rely solely on each other for support may help maintain it over time, highlighting the need for future research to explore the long-term benefits of peer-based support models. Strategies such as continued structured check-ins or digital platforms may help sustain support beyond the intervention [58].

Finally, the Actor-Partner Interdependence model indicates there was no contagion effect of physical activity between partners [31]. The minimal impact of partner MVPA may be due to the remote nature of the intervention, whereby participants were only connecting online, rather than in person. Combined with the finding that social support from the partner relates to MVPA, this suggests that the intentional support of a partner, rather than their physical activity behavior, may be a key determinant of physical activity engagement among WBC in a remote MVPA intervention. In the current study, partners were not matched through the Fitbit device and could not observe each other’s beahviors remotely. As such, sharing of physical activity behaviors was done through verbal/text information sharing (if at all) and shared behaviors may be limited [59, 60]. This study should be replicated with in-person physical activity sessions to explore contagion effects and impact of peer physical activity.

Our findings have important implications for interventions aimed at promoting physical activity among WBC. Programs targeting WBC should emphasize the importance of social support, particularly from partners, in facilitating engagement in MVPA. These strategies could include encouraging individuals to seek out support [61], educating and training partners how to provide physical activity support [62], and matching partners based on characteristics that increase the likelihood of supportive collaborations [18]. Efforts to foster a supportive environment within the WBC-partner dyad may be more impactful than focusing solely on increasing the WBC’s individual motivation or physical activity skills. Future research is needed to explore these possibilities.

There are limitations for the current study. First, fitness trackers could have influenced the physical activity behaviors of participants in both conditions [63], in particular at baseline, which may explain the discrepancy between self-report and device measured physical activity. Future studies should consider providing the Fitbit device several weeks before the intervention to allow time for individuals’ behaviors to adjust to more typical levels rather than capturing potential reactive behaviors. Further, future studies should explore methods to enhance the impact of fitness trackers while also strengthening social support, such as implementing data-sharing features for peers to observe each other’s exercise routines. The base features of the Fitbit device were masked for the duration of this study and participants could not see their partner’s data. This may also explain why the partner effects were non-significant in the final models. Additionally, the sample consisted mostly of young white WBC who were primarily in the early stages of breast cancer, and recently completed treatment. During this period, individuals with breast cancer often experience a decline in structured and healthcare support [4]. Further research is necessary to determine if these findings extend to a broader and more diverse population of individuals with breast cancer, other forms of cancer, and men as well as gender-diverse individuals. Finally, some women reported engaging in more than 150 min of MVPA at baseline. As such, these results may represent a conservative estimate of the relationship between social support and increased MVPA.

In spite of the limitations, there are many strengths to this study beyond the randomized controlled trial design. MVPA was examined through both self-report and Fitbit devices. This two-pronged approach allowed for a more comprehensive understanding of the psychological and behavioral impact of the intervention. While device measured MVPA include behaviors that participants may not perceive as physical activity (e.g., gardening, home maintenance), self-report MVPA includes only behaviors participants are aware of [64]. The effects of physical activity often depend on whether individuals perceive the behavior as healthy [54], and as such, there is significant value in understanding individuals’ subjective perceptions of their time spent in movement behaviors. The behavioral strategies delivered by the QEP were theory-informed [40] and are replicable. The partner-matching strategies were evidence-informed [39] and the analytical approach, accounting for peers in an MVPA intervention, advances understanding of the way data on MVPA align within an intervention.

Consistent with previous research [16], this study underscores the importance of social support from partners in promoting MVPA among WBC, regardless of the inclusion of a QEP. These findings highlight the importance of understanding how and when QEP involvement enhances or detracts from peer support interventions, which remains critical for optimizing physical activity promotion in this population. Further, the actual exercise behaviors of partners did not impact their own physical activity behaviors. As such, by focusing on the supportive role of partners, rather than the quantity of their physical activity, interventions can be tailored to better meet the unique needs of women post cancer diagnosis, ultimately improving their overall health and well-being.

## Electronic supplementary material

Below is the link to the electronic supplementary material.

Supplementary Material 1

Supplementary Material 2

## Data Availability

Data are available from corresponding author upon request.

## Declarations

### Full trial protocol

The full trial protocol can be reached here 10.1186/s13102-021-00341-w
