# Supplementary material for: Connecting women who are diagnosed and treated for breast cancer to engage in physical activity: a two-arm randomized controlled trial
Source: BMC Sports Sci Med Rehabil. 2025 Apr 25;17:96. doi: 10.1186/s13102-025-01131-4 (PMC12023542; doi:10.1186/s13102-025-01131-4)
Supplement: Supplementary file 2 — Supplementary Material 2 [file 13102_2025_1131_MOESM2_ESM.pdf]

Connecting BCS for Exercise RCT

LEISURE-TIME EXERCISE QUESTIONNAIRE

NOTE: In these survey questions, the term “exercise” refers to purposeful physical activity that you do to improve your physical fitness (strength, endurance, flexibility, etc.)

During a typical 7-day period (a week) in the last month, how often do you take part in the following kinds of exercise during your free time and for how long?

STRENUOUS EXERCISE - Heart beats rapidly
(e.g. running, jogging, vigorous swimming, vigorous bicycling)

Number of times per week ______________________________________
Average duration of each session (minutes) ______________________________________

MODERATE EXERCISE - Not exhausting
(e.g. fast walking, easy bicycling, easy swimming)

Number of times per week ______________________________________
Average duration of each session (minutes) ______________________________________

MILD EXERCISE - Minimal effort
(e.g. yoga, bowling, easy walking)

Number of times per week ______________________________________
Average duration of each session (minutes) ______________________________________

RESISTANCE / STRENGTH EXERCISE
(e.g., lifting weights, push ups, sit ups, resistance bands, body weight exercises)

Number of times per week ______________________________________
Average duration of each session (minutes) ______________________________________

During a typical 7-day period (a week) in the last month, in your leisure time, how often do you engage in any regular activity long enough to work up a sweat (heart beats rapidly)?

☐ Often ☐ Sometimes ☐ Never/Rarely
